# Liver Damage in *Ctenopharyngodon idellus* Induced by Nanoplastics and Cadmium Exposure

**DOI:** 10.3390/biology15131039

**Published:** 2026-06-29

**Authors:** Qifeng Gao, Jianbo Ma, Zixuan Li, Chunping Mao, Xiaodong Zhang, Chaonan Zhang

**Affiliations:** 1 College of Life and Environmental Science, Shaoxing University, Shaoxing 312000, China; 2 Yuyao Aquaculture Technology Extension Center, Ningbo 315400, China; 3 Zhejiang Provincial Key Laboratory of Organic Pollution Process and Control, College Environmental and Resource Sciences, Zhejiang University, Hangzhou 310058, China

**Keywords:** nanoplastics, Cd, *Ctenopharyngodon idellus*, liver

## Abstract

Nanoplastics (NPs) and heavy metal cadmium (Cd) are common co-existing pollutants in freshwater environments, but their combined toxic effects on the liver of herbivorous economic fish remain unclear. In this study, grass carp (*Ctenopharyngodon idella*) were exposed to polystyrene nanoplastics (PS-NPs, 100 nm) and/or Cd to investigate their individual and combined effects on hepatic toxicity. The results revealed that co-exposure interactively suppressed interleukin-10 (*IL-10*) expression and heme oxygenase-1 (*HO-1*) antioxidant response, and induced more severe hepatic necrosis, melanization, and fibrinoid necrosis, with the highest integrated biomarker response index and extensive disruption of lipid and steroid metabolism pathways. This study clarified the toxicological interaction of NPs and Cd on the liver of grass carp, and provided a theoretical basis for understanding the combined toxicity of NPs and heavy metal pollution in extreme contamination scenarios or accidental pollution events.

## Simple Summary

Nanoplastics and cadmium often occur together in freshwater, but their combined effects on fish health are not well understood. In this study, we exposed grass carp, an important food fish, to these pollutants alone or in combination for 72 h to examine liver damage. The mixture caused much more severe liver injury—including cell death, darkening, and tissue breakdown—than either pollutant alone. It also weakened the fish’s immune system and natural antioxidant defenses more strongly. By analyzing gene activity, we found that co-exposure broadly disturbed the liver’s normal fat and hormone processing. Our results show that the two pollutants together create complex harmful effects that are not simply the sum of their individual actions.

## 1. Introduction

With the continuous rise in global plastic production and the intensification of industrial activities, combined pollution from emerging and traditional environmental contaminants has become a serious challenge for aquatic ecosystems. It is estimated that without effective intervention, global plastic waste volume could reach 121 million tons by 2050 [1]. In aquatic environments, plastic products undergo continuous fragmentation due to mechanical abrasion, ultraviolet radiation, and microbial degradation, generating microplastics (MPs, <5 mm) and even nanoplastics (NPs, <1 μm) [2]. In the mixed layer of the temperate to subtropical North Atlantic, the mass of NPs may reach 27 million tons [3]. Polystyrene nanoplastics (PS-NPs) are among the most frequently detected NPs in the environment. Owing to their extremely small size, large specific surface area, and intrinsic hydrophobicity of polystyrene, PS-NPs possess a far greater capacity for pollutant adsorption and biological barrier penetration than MPs. However, it should be noted that in natural aquatic environments, the surface properties of NPs can be altered by the adsorption of humic substances and the formation of oxygen-containing functional groups due to UV irradiation, which may modify their hydrophobicity and pollutant carrier behavior [4,5]. Consequently, they readily enter aquatic organisms, cross the intestinal barrier, and accumulate in tissues such as the liver, brain, and gonads via the bloodstream [6,7].

Concurrently, heavy metal pollution cannot be ignored. Cadmium (Cd), a typical toxic heavy metal, originates from mining, electroplating, phosphate fertilizer application, urban runoff, and other sources, and is frequently detected in many water environments. For example, the estimated daily Cd intake for residents in the Ningxia section of the Yellow River Basin exceeds the WHO limit by a factor of 4.7 [8]. Cd levels in the urbanized waterways of the Pearl River Delta surpass the global average [9]. Cd is poorly metabolized and excreted in aquatic organisms, resulting in a long biological half-life (years to decades in some fish species) [10]. It accumulates mainly in the liver, kidney, and gills, and causes sustained toxicity by inducing oxidative stress, interfering with essential metal ion homeostasis, and inhibiting antioxidant enzymes and DNA repair systems [11,12,13].

In real aquatic environments, NPs and Cd co-occur and undergo complex interactions. On the one hand, the surface properties of NPs, including their surface charge and oxygen-containing functional groups, enable them to act as effective carriers for metal ions like Cd^2+^, enhancing metal bioaccumulation and toxicity transfer via the “Trojan horse” effect [14,15,16]. Li demonstrated that NPs significantly increased the uptake of Cd by hepatocyte, and the intracellular desorption of loaded Cd was the key prerequisite for amplifying cytotoxicity [17]. On the other hand, the formation of an “eco-corona” on NP surfaces or competitive adsorption may alter Cd bioavailability, potentially producing antagonistic effects [18]. Furthermore, NPs and Cd can also exert complex interactive toxicity through indirect mechanisms such as synergistic interference with signaling pathways or competition for detoxification resources [19]. Therefore, the toxic effects of combined exposure are not simply additive. Their direction and intensity depend on multiple factors, including pollutant concentration, exposure sequence, particle size, and surface properties [20,21].

The liver, as the primary detoxification organ and metabolic hub in fish, is the core target organ for NP and Cd accumulation and toxicity. Numerous studies have shown that exposure to NPs or Cd alone can induce oxidative stress, lipid metabolism disorders, immune dysregulation, and histopathological damage in fish livers [22,23,24]. Although the individual hepatotoxicity of NPs and Cd is well recognized, the effects of their combined exposure remain controversial. In Prussian carp (*Carassius gibelio*), combined exposure caused more severe hepatic necrosis and inflammation [24]. By contrast, in hybrid snakehead (*Channa maculata* ♀ × *Channa argus* ♂), combined exposure suppressed the expression of inflammatory factors [25]. This indicator-dependent divergence suggested that the combined hepatotoxicity of PS-NPs and Cd might involve both synergistic and antagonistic mechanisms, with the mode of action varying by species, exposure conditions, and endpoints, necessitating further investigation.

Grass carp (*Ctenopharyngodon idella*) is an important freshwater economic fish species in China. Because of its wide distribution and sensitivity to pollutants, it is often used as a model organism in aquatic toxicology [26]. Previous studies have shown that grass carp are highly sensitive to NPs and heavy metal exposure, with the liver being a major target organ [27,28]. However, most studies on grass carp have focused on the single toxicity of NPs or Cd, and systematic reports on the multi-level toxic effects and mechanisms of their combined exposure on the grass carp liver are lacking. Moreover, existing combined toxicity studies have often focused on single dimensions such as biochemical parameters or gene expression, lacking multi-scale integration from molecular pathways to histopathology. By combining omics analysis with traditional toxicological methods, this study aimed to provide a deeper understanding of the toxicity mechanisms of PS-NPs and Cd co-exposure on the grass carp liver from both macroscopic phenotypes and molecular networks. The results would elucidate the joint toxicity of NPs and Cd on aquatic organisms under high-exposure conditions, thereby offering a methodological reference for future research focused on environmentally relevant concentrations and long-term exposure in freshwater aquaculture environments.

## 2. Materials and Methods

### 2.1. Materials

Fluorescent polystyrene nanoparticles (PS-NPs, PS-7-3-0010) with a mean diameter of 100 nm were supplied by BaseLine ChromTech Research Centre (Tianjin, China). According to the manufacturer, the particles are plain polystyrene with no charged surface groups. The nanoparticles are stabilized by anionic surfactants, resulting in a negatively charged surface (zeta potential: −16.6 ± 1.8 mV, as reported by the manufacturer). The fluorescence excitation/emission peaks are 518/458 nm. A stock suspension (10 mg/mL) was stored at 4 °C in the dark and vigorously shaken before each use to ensure uniform dispersion. These nanoparticles have been widely employed in nanotoxicological studies [29,30]. Cadmium chloride (CdCl_2_, CAS 10108-64-2, AR ≥ 99.99%) was purchased from Shanghai Aladdin Technology Co., Ltd. (Shanghai, China). Mixed solutions were prepared by diluting the PS-NP and CdCl_2_ stock solutions with deionized water.

Healthy grass carp juvenile were obtained from a local aquaculture farm in Huzhou City (Zhejiang, China) and transported to the laboratory in oxygen-filled bags. Their body length was 5.87 ± 0.02 cm and their weight was 3.99 ± 0.13 g. The fish were placed in the 100 L glass tank with continuous aeration at ambient temperature (~26 °C) and allowed to acclimate for two weeks. During this period, the fish were fed a commercial diet twice daily. Feeding was withheld for 48 h before the exposure trial to standardize their metabolic condition.

### 2.2. Experiment Set

Acute exposure trials were performed to elucidate the damage mechanisms of the two contaminants in fish. The concentrations of NPs (1 mg/L) was selected based on a comprehensive review of the existing literature and environmental monitoring data [31,32,33]. The Cd concentration of 1 mg/L was selected based on preliminary toxicity tests and published data on Cd toxicity in grass carp [34,35].

Feeding was suspended 48 h before the experiment. Grass carp were randomly assigned to 12 glass aquaria (35 cm × 20 cm × 22 cm) at a density of 10 fish per aquarium. The randomly arranged aquaria were divided into four groups with three replicates each, giving 30 fish per treatment. The control group (CK) was kept in tap water. The NPs group was exposed to 1 mg/L 100 nm PS-NPs, the Cd group received 1 mg/L CdCl_2_, and the NPs + Cd group was co-exposed to a mixture of 1 mg/L CdCl_2_ and 1 mg/L 100 nm PS-NPs. All experimental procedures involving fish were approved and supervised by the Committee for the Welfare and Ethics of Laboratory Animals of Shaoxing University (NO. 20250531). After 72 h of exposure, the fish were collected from each aquarium and dissected on ice to obtain liver tissues. Some tissue samples were rapidly processed with liquid nitrogen and stored at −80 °C for further analyses. Fresh samples preserved in 4% PFA fixative (BL539A, Anhui BaiSha Biotechnology Co., Ltd., Baisha, China) were used for histopathological section analysis and could be stored at room temperature after fixation.

### 2.3. Analyses of Biochemical Parameters

Liver samples were homogenized in nine volumes of ice-cold homogenization medium (0.86% normal saline) using a high-speed tissue homogenizer (KZ-II, Servicebio, Wuhan, China) in an ice-water bath to yield 10% (*w*/*v*) homogenates. The homogenates were then centrifuged at 2500 rpm for 10 min at 4 °C in a high-speed refrigerated centrifuge (D3024R, Dalongxingchuang Experimental Instruments (Beijing) Co., Ltd., Beijing, China), and the supernatants were retained for measurement. The activities of superoxide dismutase (SOD), catalase (CAT), and lactate dehydrogenase (LDH), as well as the content of glutathione S-transferase (GSH-ST), were determined using commercial reagent kits from Nanjing Jiancheng Institute of Biological Engineering (Nanjing, China) on a multimode microplate reader (Infinite M200 PRO, Tecan, Männedorf, Switzerland).

### 2.4. Analysis of Gene Expression Levels

*IL-1β*, *IL-8* and *IL-10* were selected as typical representatives of immunomodulatory factors, and *MT-2*, *HO-1*, and *ZO-1* were selected as representatives of oxidative stress and tight junction integrity markers. The expression differences in these genes in liver tissues of each group were also analyzed. Specific primer sequences are listed in Table S1 in the Supporting Information (SI) [35,36,37,38]. Details of methods for RNA extraction, cDNA synthesis, and the real-time PCR program settings are presented in Text S1.

### 2.5. Histopathological Analyses

The fresh liver tissues were fixed in general-purpose tissue fixator (Servicebio, Wuhan, China), embedded in paraffin wax, sectioned at 4 μm thickness, and stained with hematoxylin–eosin (H&E). Tissue slices were examined and photographed by a microscope (Eclipse 80i, Nikon, Tokyo, Japan) with the microscopic image analysis software (ImageView 4.1).

### 2.6. Transcriptome Sequencing

RNA extraction, RNA purification, reverse transcription, library construction, and sequencing were performed at Shanghai Majorbio Bio-pharm Biotechnology Co. (Shanghai, China) according to the manufacturer’s instructions (Illumina, San Diego, CA, USA). Detailed methods are described in Text S2.

### 2.7. Statistical Analyses

Statistical analyses were performed with IBM SPSS Statistics v19.0 by the one-way analysis of variance (ANOVA) after verification of the normality and variance. A significance level of *p* < 0.05 indicated statistical significance. The expression profiles among the four groups were analyzed by ANOVA with Tukey test. Data visualization was carried out using GraphPad Prism 9.0.0 (San Diego, CA, USA).

## 3. Results

### 3.1. Changes in Hepatic Biochemical Parameters

**Figure 1:**
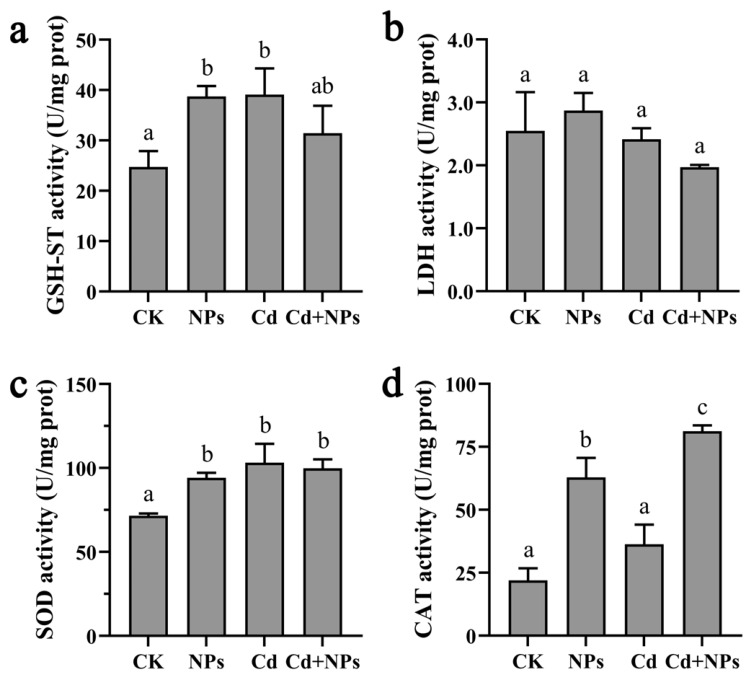
Analyses of biochemical parameters in the liver tissue of grass carp after the 72 h exposure. (**a**) GSH-ST activity, (**b**) LDH activity, (**c**) SOD activity, (**d**) CAT activity. Data are expressed as mean ± SD (*n* = 3). a,b,c Mean values within a row without a common superscript letter were significantly different (*p* < 0.05).

After 72 h of exposure, the activities of GSH-ST, LDH, SOD, and CAT in the liver of grass carp were measured. Compared with the control group, GSH-ST activity was significantly increased in both the NPs group and the Cd group. Although the combined exposure group also exhibited an upward trend, no significant difference was observed relative to the control group (Figure 1a). LDH activity remained generally stable across all treatment groups, with no significant intergroup differences (Figure 1b). For SOD, the activities in the three experimental groups were all significantly higher than that in the control group, but no significant differences were detected among the experimental groups, indicating that both single and combined exposure activated SOD expression to a similar extent (Figure 1c). CAT activity exhibited a different pattern: it was significantly elevated in both the NPs group and the NPs + Cd group, with a greater increase in the NPs + Cd group than in the NPs group, suggesting that NPs may have played a dominant role in inducing CAT activity (Figure 1d).

### 3.2. Analyses of Relative Expression Levels of Immune Genes

**Figure 2:**
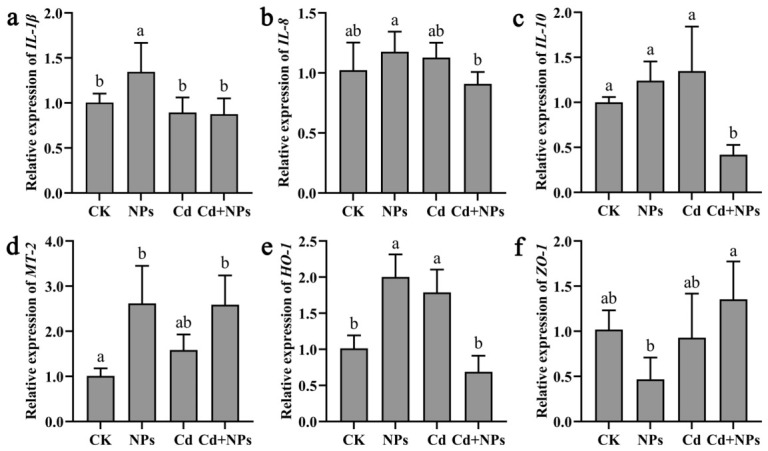
Indicators in the liver of grass carp after the 72 h exposure. (**a**) *IL-1β*, (**b**) *IL-8*, (**c**) *IL-10*, (**d**) *MT-2*, (**e**) *HO-1*, and (**f**) *ZO-1*. Data are expressed as mean ± SD (*n* = 3). a,b Mean values within a row without a common superscript letter were significantly different (*p* < 0.05) through the ANOVA with Tukey test.

By analyzing the relative expression levels of *IL-1β*, *IL-8*, *IL-10*, *MT-2*, *HO-1*, and *ZO-1* in the liver, the distinct immune responses were observed. The expression of the pro-inflammatory cytokine *IL-1β* was significantly upregulated after NP exposure alone, reaching the highest level among all groups. However, this strong pro-inflammatory response was not observed in the NPs + Cd group (Figure 2a). For the chemokine *IL-8*, its expression was relatively high in the NPs group, whereas the combined exposure significantly attenuated this upregulation (Figure 2b). The expression of the anti-inflammatory cytokine *IL-10* was specifically suppressed under combined exposure conditions, with its expression level significantly lower than those in the control group and the other exposure groups (Figure 2c). The expression of the heavy metal detoxification marker *MT-2* was significantly upregulated in both the NPs group and the NPs + Cd group, markedly exceeding that in the control group (Figure 2d). In terms of antioxidant defense, *HO-1* exhibited significant upregulation after individual exposure to either NPs or Cd, but this response was significantly attenuated after combined exposure, with the expression level approaching that of the control group (Figure 2e). The expression of the tight junction protein *ZO-1* in the combined exposure group was significantly higher than that in the NPs group, but showed no significant difference compared with the control group and the Cd group (Figure 2f).

### 3.3. Histopathological Observations

**Figure 3:**
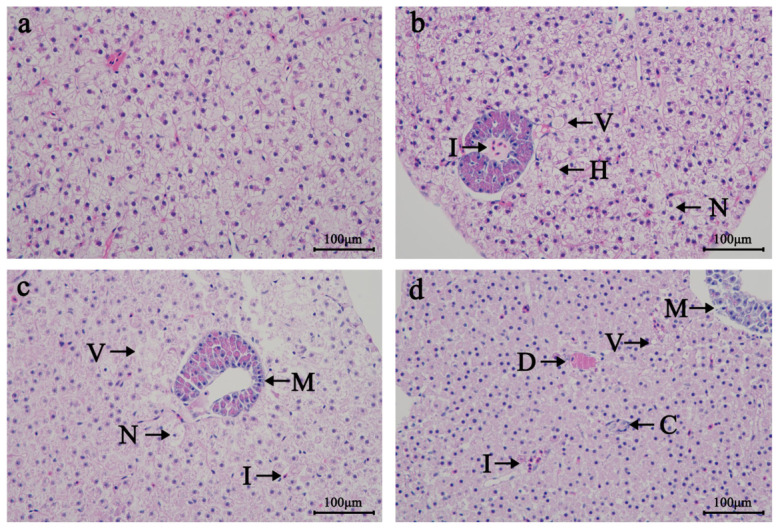
Histopathological analyses in the (**a**) control, (**b**) NPs, (**c**) Cd, and (**d**) NPs + Cd groups. (H) hepatocyte hypertrophy, (I) hemocyte infiltration, (V) vacuolization, (M) melanism, (C) cell necrosis and (D) diffuse fibrinoid necrosis of ellipsoidal sheath.

Histopathological analysis showed that vacuolization and hemocyte infiltration were observed in all experimental groups. Hepatocyte hyperplasia was found in the NP exposure group. Melanism was observed in both the Cd exposure group and the combined exposure group. In the combined exposure group, cell necrosis and diffuse fibrinoid necrosis of the ellipsoidal sheath were also detected. Compared with the control group, the NP exposure group exhibited relatively intact cell morphology, whereas the Cd and combined exposure groups displayed cell fragmentation, and even melanism and necrosis (Figure 3).

### 3.4. Transcriptome Sequencing Analyses

**Figure 4:**
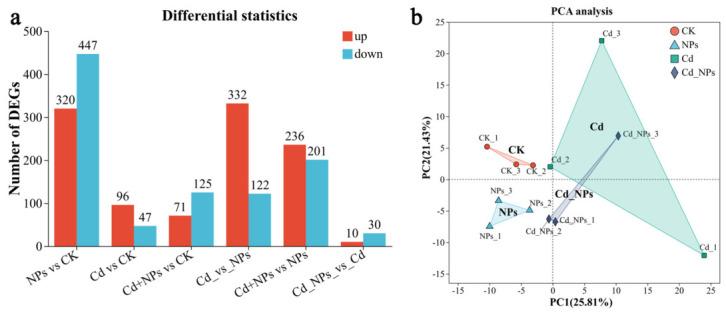
(**a**) Statistical chart of differential expression. (**b**) PCA plot of samples.

Transcriptome sequencing analyses were conducted on twelve liver tissue samples from four groups, resulting in a total of 73.21 Gb of clean data. The high mapped ratios (93.47–96.71%) observed in all samples confirm the absence of contamination and attest to the high fidelity of the sequencing data, thereby ensuring their suitability for all downstream analyses (Table S2). A total of 2037 differentially expressed genes (DEGs) were identified, among which 1065 were upregulated genes and 972 were downregulated genes (Figure 4a). Principal component analysis (PCA) of gene profiles among different groups demonstrated that the samples from the control, the NPs, and the NPs + Cd groups exhibited a clear separation trend on the plane defined by PC1 and PC2, indicating significant differences in the gene expression profiles among the three groups (Figure 4b).

**Figure 5:**
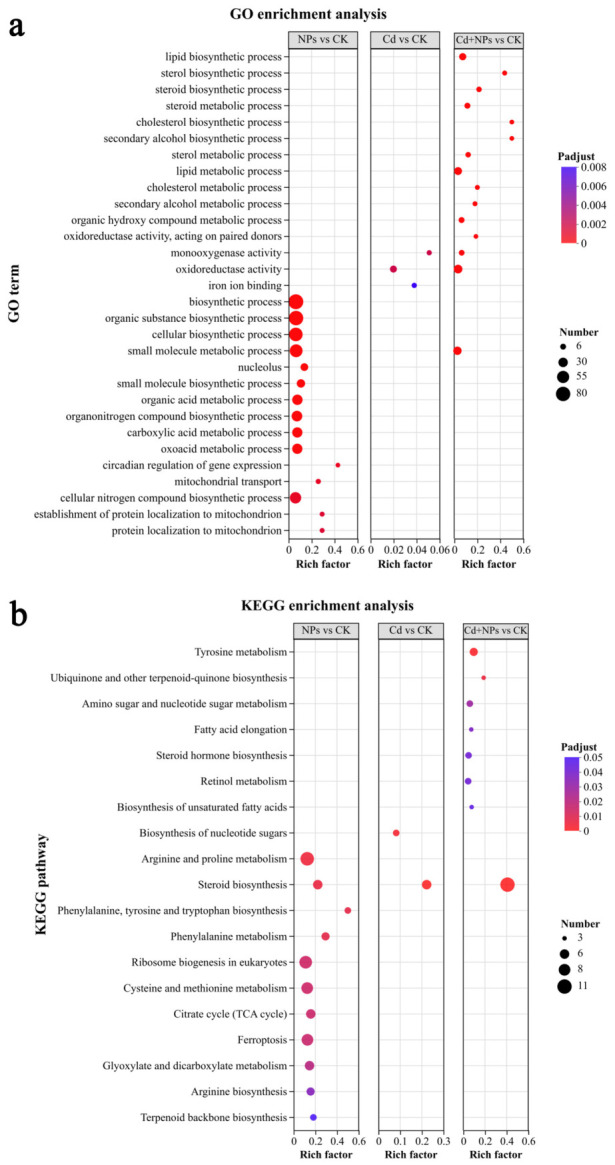
(**a**) GO enrichment analysis comparing each exposure group with the control group. (**b**) KEGG enrichment analysis comparing each exposure group with the control group.

Gene ontology (GO) and Kyoto encyclopedia of genes and genomes (KEGG) enrichment analyses reveal the biological functions and underlying key pathways of the DEGs. The three DEG sets (NPs vs. CK, Cd vs. CK and NPs + Cd vs. CK) were shown the thirty most enriched GO terms. The NPs + Cd group was significantly enriched in “lipid biosynthetic process”, “sterol biosynthetic process”, and “cholesterol biosynthetic process”. The differentially expressed genes in the Cd group were significantly enriched in “monooxygenase activity”, “oxidoreductase activity”, and “iron ion binding” items. The NPs group were significantly enriched in “small molecule metabolic process”, “organic substance biosynthetic process”, “cellular nitrogen compound biosynthetic process”, and “nucleolus” items (Figure 5a).

Compared with the CK group, the main enriched KEGG pathway in the NPs + Cd group mainly activated eight pathways, including “steroid biosynthesis”, “tyrosine metabolism”, “ubiquinone and other terpenoid-quinone biosynthesis”, and “amino sugar and nucleotide sugar metabolism” (Figure 5b).

### 3.5. The IBR Index and the Abbott Model

**Figure 6:**
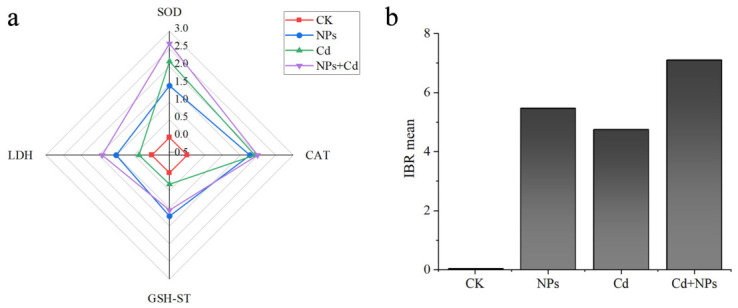
(**a**) Radar chart showing the score values (S_i_) of each biochemical parameter. (**b**) IBR values of each treatment group.

Four biomarkers—SOD, CAT, GSH-ST, and LDH—were selected. Based on the degree of inhibition or activation of their activities, the integrated biomarker response (IBR) index was calculated to comprehensively evaluate the toxic stress exerted by NPs and Cd on the grass carp liver. Detailed calculation steps were based on Ganie et al. and shown in Text S3 [39]. The IBR index ranked as follows: NPs + Cd group > NPs group > Cd group > CK group, intuitively reflecting that the combined exposure caused the most severe biological damage (Figure 6).

## 4. Discussion

### 4.1. Response of Antioxidant Enzyme Activities and Characteristics of Combined Toxicity

Antioxidant enzyme activities are classical indicators reflecting oxidative stress induced by environmental pollutants. In the present study, SOD activity was significantly elevated in all groups, with no significant difference among the groups, indicating that both PS-NPs and Cd could independently induce oxidative stress and that no obvious interactive effect was observed for this indicator. This result was consistent with previous studies. Short-term exposure to low concentrations of Cd could induce increased SOD activity [40], and PS-MPs have been demonstrated to induce oxidative stress in fish liver [41]. In contrast, CAT activity was significantly higher in the NPs group and the NPs + Cd group than in the Cd group and the control group, suggesting that PS-NPs played a dominant role in CAT induction. In the co-exposure group, GSH-ST and LDH activities showed no significant differences compared with the single exposure groups, suggesting an antagonistic interaction at the biochemical level. Similar antagonistic effects were also reported by Wen et al. in discus fish (*Symphysodon aequifasciatus*), who found that co-exposure to MPs and Cd resulted in antagonistic interactions for several biochemical parameters (catalase, acid phosphatase, alkaline phosphatase, and complement 3), yet also produced synergistic increases in protein carbonyl content and lysozyme activity, ultimately inducing severe oxidative stress and stimulating innate immunity [42]. Importantly, the combined effects of PS-NPs and Cd were not uniformly synergistic across all endpoints; while some biochemical responses (GSH-ST and LDH) exhibited antagonistic or additive patterns, histopathological damage and transcriptomic disruptions (particularly in lipid and steroid metabolic pathways) were clearly aggravated. This endpoint-dependent nature underscores that the overall toxicity of combined exposure should be viewed as a complex integration of multiple, sometimes opposing, interaction mechanisms, and evaluating such toxicity therefore requires integrating multiple endpoints across different biological scales rather than relying on a limited set of enzyme activities. Histopathological observations further supported this notion. The co-exposure group exhibited severe lesions such as hepatocyte necrosis, melanization, and diffuse fibrinoid necrosis, whereas the single exposure groups showed relatively milder damage. This suggested that although some enzymatic indicators present antagonism, the tissue-level damage tends to be exacerbated, which might be related to metabolic disturbances, ferroptosis, or activation of apoptotic pathways induced by co-exposure [43].

### 4.2. Co-Exposure Specifically Suppresses Anti-Inflammatory Immunity and Disrupts Immune Homeostasis

The expression changes in immune-related genes revealed a non-additive interference of co-exposure with the host immune system. *IL-1β* was significantly upregulated in the NPs group, consistent with existing studies showing that NPs induced pro-inflammatory responses through the NF-κB pathway [44]. However, this upregulation was suppressed in the NPs + Cd group, which might be attributed to Cd interference with the TLR4-NF-κB signaling pathway or the induction of immune cell apoptosis [45]. More importantly, *IL-10* was significantly downregulated only in the NPs + Cd group. As *IL-10* is a core molecule of the anti-inflammatory response, its downregulation suggested that co-exposure triggers a severe imbalance in immune homeostasis. Studies have shown that combined stress could inhibit IL-10 transcription through DNA methylation or STAT3 signaling [46]. Meanwhile, *MT-2* was significantly upregulated in both the NPs and NPs + Cd groups, reflecting a compensatory protective response of the organism to metal imbalance and physical damage via the Nrf2/MTF-1 pathway [47]. *HO-1* was significantly upregulated in the single exposure groups but lost its responsiveness in the co-exposure group. This pattern might suggest a disruption of the coordinated antioxidant/anti-inflammatory response, although alternative explanations such as transcriptional inhibition of *HO-1* under severe stress or a shift toward other stress pathways (e.g., ferroptosis, apoptosis) cannot be ruled out [48]. NPs might cause direct physical damage to the intestinal or gill barrier, leading to the downregulation of *ZO-1*. Co-exposure could activate the Wnt/β-catenin pathway, triggering compensatory tight junction protein synthesis to cope with more severe barrier disruption [49].

### 4.3. Transcriptomic Analysis Reveals Widespread Disruption of Lipid and Steroid Metabolism upon Co-Exposure

Transcriptomic analysis showed that, compared with the control group, the differentially expressed genes in the PS-NPs and Cd co-exposure group were significantly enriched in GO terms related to lipid, sterol and steroid biosynthetic and metabolic processes, and KEGG pathways pointed to widespread perturbations in ubiquinone synthesis, steroid hormone synthesis, retinol metabolism, unsaturated fatty acid synthesis and elongation. None of these pathways were as intensively or significantly enriched in the NPs or Cd alone groups, suggesting that the co-exposure non-additively and systemically disrupted the hepatic lipid metabolic network in grass carp.

The enrichment of the “biosynthesis of unsaturated fatty acids” and “fatty acid elongation” pathways in the co-exposure group suggests that the liver may attempt to remodel fatty acid chain length and unsaturation in response to endoplasmic reticulum stress or altered membrane fluidity. Previous studies have shown that nanoplastics can activate sterol regulatory element-binding proteins (SREBPs) to induce aberrant lipid accumulation [41,50]. Additionally, Cd can cause the deposition of lipid droplets (LD) in the nuclei of liver cells, thereby leading to lipid metabolism disorders [51]. When the two pollutants are combined, crosstalk between these signalling pathways likely underlies the extensive lipid metabolic disturbance. The enrichment of the “steroid hormone biosynthesis” pathway is particularly noteworthy, as steroid hormones are key effector molecules of the hypothalamic–pituitary–interrenal (HPI) axis in fish. Dysregulation of this pathway raised the possibility of interference with neuroendocrine stress responses, providing a new endocrine perspective to explain the significant downregulation of *IL-10* and tissue necrosis observed in the NPs + Cd group [52,53]. Nevertheless, direct measurements of lipid and hormone levels are required to validate these functional alterations. In addition, the alteration in retinol metabolism reflects disturbed hepatic vitamin A homeostasis, which may weaken hepatocyte barrier repair capacity and exacerbate histopathological damage [53]. The enrichment of the ubiquinone biosynthesis pathway may indicate a compensatory response to mitochondrial electron transport chain dysfunction, suggesting that co-exposure induces an energy metabolism crisis [54]. The significant enrichment of GO terms related to “oxidoreductase activity” and “monooxygenase activity” further suggests activation of cytochrome P450 enzymes to accelerate xenobiotic metabolism, but the reactive oxygen species generated during this process may aggravate oxidative stress [55,56]. It is important to emphasize that transcriptomic enrichment analysis indicates potential molecular perturbations, but does not directly measure functional outcomes, such as lipid accumulation, triglyceride or cholesterol levels, or hormone concentrations. Taken together, the toxicity of PS-NPs and Cd co-exposure to grass carp liver is characterized at the molecular level by widespread disruption of lipid and steroid homeostasis, which may contribute to the severe liver injury observed in histopathology. It should be noted that transcriptomic changes indicated pathway enrichment, but did not prove functional alteration. All mechanistic interpretations are presented as hypotheses requiring experimental validation.

## 5. Conclusions

The present study revealed the effects of single and combined exposures to NPs and Cd on the liver of grass carp. Combined exposure significantly suppressed *IL-10* and *HO-1*, while causing more severe hepatic necrosis, melanization, and fibrinoid necrosis. The IBR index was highest in the co-exposure group. Transcriptomic analysis further revealed that co-exposure broadly disrupted lipid, sterol, and steroid metabolic pathways, providing a molecular basis for the observed histopathological damage. However, direct measurements of lipid and hormone levels are needed to confirm these functional changes. Overall, PS-NPs and Cd exhibited endpoint-dependent interactions in grass carp liver, with aggravated hepatotoxicity at histopathological and transcriptomic levels despite some biochemical parameters showing antagonistic or additive effects. This indicated that the combined toxicity was not simply synergistic but rather multi-faceted and endpoint-specific. Future studies should emphasize long-term chronic exposure experiments to further elucidate the molecular regulatory networks and ecological health risks of combined NP and heavy metal pollution.

## Data Availability

All data related to this study are contained within the manuscript. Data can be obtained from the corresponding authors on request.

## Supplementary Materials

The following supporting information can be downloaded at: https://www.mdpi.com/article/10.3390/biology15131039/s1. Text S1. Details of methods for qPCR; Text S2. The method of transcriptome sequencing; Text S3. The calculation method of IBR index; Table S1. Sequences of primers used for qPCR; Table S2. Transcriptome quality control form.
