# Supplementary material for: Liver Damage in Ctenopharyngodon idellus Induced by Nanoplastics and Cadmium Exposure
Source: Biology (Basel). 2026 Jun 29;15(13):1039. doi: 10.3390/biology15131039 (PMC13360025; doi:10.3390/biology15131039)
Supplement: Supplementary file 1 [file biology-15-01039-s001.zip › biology-4384045-supplementary.pdf]

Supporting information

Liver Damage in *Ctenopharyngodon idellus* Induced by Nanoplastics and Cadmium Exposure

Qifeng Gao [1,†], Jianbo Ma [2,†], Zixuan Li [1], Chunping Mao [1], Xiaodong Zhang [1,*] and Chaonan Zhang [1,3,*]

1 College of Life and Environmental Science, Shaoxing University, Shaoxing 312000, China
2 Yuyao Aquaculture Technology Extension Center, Ningbo 315400, China
3 Zhejiang Provincial Key Laboratory of Organic Pollution Process and Control, College Environmental and Resource Sciences, Zhejiang University, Hangzhou 310058, China
* Correspondence: 13676885816@163.com (X.Z.); zhangchaonan@usx.edu.cn (C.Z.)
† These authors contributed equally to this work.

Text S1. Details of methods for qPCR

Total RNA was isolated using the MiniBEST Universal RNA Extraction Kit (Takara, Japan) in accordance with the manufacturer's protocol. RNA integrity was assessed by electrophoresis on a 1.0% agarose gel, and its concentration was measured with a Micro High Precision Spectrophotometer (NanoDrop One, Thermo Scientific, USA). First-strand cDNA was synthesized from the extracted RNA with the Magic 1st cDNA Synthesis Kit (Magicbio, China) following the manufacturer's instructions, and the resulting cDNA was stored at -80 °C for further analysis.

Quantitative real-time PCR (qRT-PCR) was conducted using a SYBR Green-based detection approach. Reactions were assembled with Magic SYBR Green qPCR Mix (Magicbio, China) and run on a CFX Connect Real-Time PCR Detection System (BIO-RAD, USA). The two-step thermal cycling program consisted of an initial pre-denaturation at 95 °C for 5 min, followed by 40 cycles of denaturation at 95 °C for 10 s and annealing/extension at 60 °C for 30 s. Melt curve analysis was subsequently carried out by heating from 65 °C to 95 °C at a ramp rate of 0.5 °C/s to monitor the dissociation of double-stranded DNA. All amplification reactions were performed with three biological replicates. At the end of the run, cycle threshold (Ct) values were collected for each sample, and the relative expression levels of target genes were calculated by the $2^{-\Delta\Delta Ct}$ method.

Text S2. The method of transcriptome sequencing.

Following a 96-h exposure period, total RNA was isolated from the four groups of juvenile test fish using TRIzol® reagent as instructed by the manufacturer (Invitrogen, USA), and any contaminating genomic DNA was digested with DNase I (TaKaRa, Japan). RNA integrity and purity were assessed with a 2100 Bioanalyzer (Agilent Technologies, Germany), and the RNA concentration was measured on a NanoDrop™ 2000 spectrophotometer (Thermo Scientific, USA). Only RNA preparations that met the following quality thresholds were used for library construction: OD260/280 ranging from 1.8 to 2.2, OD260/230 $\geqslant$ 2.0, RNA integrity number (RIN) $\geqslant$ 8.0, a 28S:18S rRNA ratio $\geqslant$ 1.0, and a total quantity exceeding 1 μg.

Transcriptome libraries were generated with 1 μg of total RNA each, utilizing the TruSeq™ RNA Sample Preparation Kit (Illumina, San Diego, USA). Polyadenylated mRNA was captured by oligo(dT) magnetic beads and subsequently fragmented in fragmentation buffer. From these fragments, double-stranded cDNA was synthesized with the SuperScript Double-Stranded cDNA Synthesis Kit (Invitrogen, USA) and random hexamer primers. In line with Illumina's standard procedure, the cDNA then underwent end-repair, phosphorylation, and A-tailing. The resulting cDNA molecules were size-fractionated on a 2% Low Range Ultra agarose gel, and fragments centered around 300 bp were excised and recovered. Fifteen cycles of PCR amplification were carried out using Phusion DNA polymerase (NEB). After the amplified libraries were quantified with a TBS380 fluorometer and passed quality control, paired-end sequencing (2 × 150 bp) was performed on the Illumina NovaSeq 6000 platform.

Raw paired-end reads were filtered and trimmed with fastp (https://github.com/OpenGene/fastp) under default settings to remove adapters, low-quality reads, and contaminants, yielding high-quality clean data. These clean reads were then de novo assembled using Trinity (http://trinityrnaseq.sourceforge.net/) to reconstruct the transcriptome. The completeness and redundancy of the assembly were evaluated and optimized through BUSCO (http://busco.ezlab.org), TransRate (http://hibberdlab.com/transrate/), and CD-HIT (http://weizhongli-lab.org/cd-hit/). To assign functional annotations, all assembled transcripts were searched by BLASTX against the NCBI non-redundant protein (NR, ftp://ftp.ncbi.nlm.nih.gov/blast/db/), Swiss-Prot (http://web.expasy.org/docs/swiss-prot_guideline.html), Pfam (http://pfam.xfam.org/), COG

(http://www.ncbi.nlm.nih.gov/COG/), GO (http://www.geneontology.org), and KEGG (http://www.genome.jp/kegg/) databases, using an E-value cutoff of < 1.0×10^{-5}; the best hits were retrieved together with their functional descriptions.

For differential expression analysis, transcript abundance was quantified by RSEM (http://deweylab.biostat.wisc.edu/rsem/) and normalized as transcripts per million (TPM). Differentially expressed genes (DEGs) between groups were identified with DESeq2, DEGseq, edgeR, Limma, or NOIseq. Significance thresholds were set at $|\log_2(\text{fold change})| \geqslant 1$ and an adjusted P (P-adjust) $\leqslant$ 0.05 for DESeq2/edgeR/Limma, P-adjust $\leqslant$ 0.001 for DEGseq, or a probability > 0.8 for NOIseq. All unigenes were aligned to GO and KEGG databases to derive functional information. DEG functional enrichment analyses, including GO term and KEGG pathway enrichment, were conducted with Goatools (https://github.com/tanghaibao/Goatools) and KOBAS (http://kobas.cbi.pku.edu.cn/home.do) using the whole transcriptome as the reference background, and terms or pathways with corrected P $\leqslant$ 0.05 were deemed significantly enriched.

Text S3. The calculation method of IBR index.

The calculation method was based on the article by Ganie et al.[1].

For each type of biomarker in the various treatments, its average value (X_i) is standardized using the overall average value (m) and standard deviation (s) of all the treatments:

$$\boldsymbol{Y_i = \frac{X_i - m}{s}} \qquad ①$$

Based on whether the biological reaction is activating or inhibiting, multiply each standardized value (Y_i) by 1 or -1:

$$\boldsymbol{Z_i = Y_i \ or\ Z_i = -Y_i} \qquad ②$$

For each biomarker in each processing method, the score value (S_i) is obtained by adding the absolute minimum value of Z_i to the Z_i value:

$$\boldsymbol{S_i = Z_i + |min|} \qquad ③$$

The S_i values of all biomarkers under each treatment method are plotted on a radar chart. Then, the IBR value is calculated based on the following formula, which is the total area of the radar chart:

$$\boldsymbol{A_i = \frac{S_i \times S_{i+1} \times \sin\left(\frac{2\pi}{k}\right)}{2}} \qquad ④$$

$$\boldsymbol{IBR = \sum_{i=1}^{k} A_i} \qquad ⑤$$

There, *k* represents the number of the biomarkers being measured.

Table S1. Sequences of primers used for qPCR

Gene	Primer Sequences (5’ → 3’)
β-actin[2]	Forward: GGCTGTGCTGTCCCTGTA
	Reverse: TTATTGTGGTTACGCTGGA
IL-1β[2]	Forward: AGAGTTTGGTGAAGAAGAGG
	Reverse: TTATTGTGGTTACGCTGGA
IL-8[2]	Forward: ATGAGTCTTAGAGGTCTGGGT
	Reverse: ACAGTGAGGGCTAGGAGGG
IL-10[2]	Forward: CTCCGTTCTGCATACAGAGAAA
	Reverse: TCATGACGTGACAGCCATAAG
MT-2[3]	Forward: ATGGATCCTTGCGACTGCG
	Reverse: CATTGACAGCAGCTGGAGCC
HO-1[4]	Forward: ACATGCCTATACACGCTATCTCG
	Reverse: CGTCACTCCAGGAAATGAGAAGA
ZO-1[5]	Forward: CGGTGTCTTCGTAGTCGG
	Reverse: CAGTTGGTTTGGGTTTCAG

Table S2. Transcriptome quality control form

Group	Sample	Total reads	Total mapped	Mapped ratio
Control group	CK-1	40070854	38718927	96.63%
	CK-2	37335454	35357964	94.7%
	CK-3	43442650	41977326	96.63%
NPs exposure group	NPs-1	41833364	40264056	96.25%
	NPs-2	40656568	39254145	96.55%
	NPs-3	36100550	34895155	96.66%
Cd exposure group	Cd-1	42237528	39477338	93.47%
	Cd-2	42383922	40990042	96.71%

	Cd-3	42019376	40303955	95.92%
Cd+NPs exposure group	Cd+NPs-1	41825326	40084306	95.84%
	Cd+NPs-2	42628926	40859238	95.85%
	Cd+NPs-3	38015000	36479735	95.96%

References

1. Ganie, Z.A.; Guchhait, S.; Talib, M.; Choudhary, A.; Darbha, G.K. Investigating the Sorption of Zinc-Oxide Nanoparticles on Tire Wear Particles and Their Toxic Effects on Chlorella Vulgaris: Insights from Toxicological Models and Physiological Analysis. *J. Hazard. Mater.* **2025**, *483*, doi: 10.1016/j.jhazmat.2024.136648.
2. Li, Z.; Yan, L.; Junaid, M.; Chen, X.; Liao, H.; Gao, D.; Wang, Q.; Zhang, Y.; Wang, J. Impacts of Polystyrene Nanoplastics at the Environmentally Relevant and Sub-Lethal Concentrations on the Oxidative Stress, Immune Responses, and Gut Microbiota to Grass Carp (ctenopharyngodon Idella). *J. Hazard. Mater.* **2023**, *441*, doi: 10.1016/j.jhazmat.2022.129995.
3. Tan, S.; Li, H.; Jin, Y.; Yu, H. Cadmium Exposure Affects on the Expression of Metallothionein 2 Gene in Grass Carp (Ctenopharyngodon Idellus). *Genes Genom.* **2016**, *38*, doi: 10.1007/s13258-015-0347-5.
4. Qin, J. Effects of Microcystin-LR on the Microstructure of Tissues and HO-1,IL-10R1 Gene Expression of Grass Carp(Ctenopharyngodon idella). *Chinese Journal of Cell Biology* **2023**, *45*, 1161-1172, doi: 10.11844/cjcb.2023.08.0003.
5. Zhang, Y.; Duan, X.; Feng, L.; Jiang, W.; Wu, P.; Liu, Y.; Kuang, S.; Tang, L.; Zhou, X. Soybean Glycinin Disrupted Intestinal Structural Integrity Related to Aggravation of Apoptosis and Downregulated Transcription of Tight Junction Proteins in the Intestine of Juvenile Grass Carp (ctenopharyngodon Idella). *Aquaculture* **2021**, *531*, doi: 10.1016/j.aquaculture.2020.735909.
